# Homœopathy

**Published:** 1884-10

**Authors:** Frank S. Billings


### THE JOURNAL

OF

### COMPARATIVE MEDICINE AND SURGERY.

VOL. V. OCTOBER, 1884. No. 4.

### ORIGINAL COMMUNICATIONS.

### ART. XXII.—HOMŒOPATHY.

*A Contribution to the Code Question.*

BY FRANK S. BILLINGS, V.S.

The "Code Question," as it has been called, has excited more or less feeling and interest, not only in New York, but in the medical profession all over the land. We have also taken some interest in reading the various articles, as well as reports of discussions, that have been written upon it. These have led us to give some thought to the subject.

It may be asked, "What has a veterinarian to do with questions that have no especial bearing upon his profession, or its work, but belong exclusively to human medicine?" It is with the hope of opening the doors of this erroneous spirit that we have undertaken this subject. A diligent study of both human and veterinary medicine, has most conclusively taught us that there is scarcely a subject which the members of either profession can discuss that has not some interest to the other. It would certainly have been to the interest of the medical, and to the advantage of the veterinary profession, had this fact received fuller recognition. The great tree which we will call "Medical Science," has but one trunk—its roots are all the same, and derive their nourishment from a common fountain—which soon divides into two great stems—*Human* and *Veterinary Medicine.* These have again been united under the name of *Comparative Medicine.* To call veterinary medicine, or any of its parts, "comparative," is a misnomer. Comparative anatomy is the comparison of all the results of anatomical research that have been made upon the highest and lowest forms of animal life, and to some extent, of vegetable also, one with another. Any other form of anatomy is special in its nature. Hence, as an earnest student of comparative medicine, we claim the right of discussing any topic of interest, whether it may have originated in human or veterinary medicine. This right has been denied us, as a veterinarian, by the Editor of the *Medical and Surgical Reporter* of Philadelphia.

One of the strongest arguments that can possibly be advanced against the validity of homœopathy is the fact, which cannot be disputed, that it has never been adopted by the veterinary profession. There has never been a single veterinarian, and there is not one living that has ever enjoyed a noteworthy reputation, either as author or practitioner, who was known as a homœopath. It has never made any impression upon any of the great veterinary schools of the world, notwithstanding many experiments with its peculiarly prepared remedies.

In this connection let us anticipate a little. The therapeutical principle of homœopathy is centred in the words "Similia similibus curantur," which, according to the orthodox of this sect in medicine, mean that "in order to cure one disease in a given organism, a so-called medicinal disease must be produced having similar symptoms"; i. e., two diseases would be raging in one and the same body at one and the same time. According to homœopathy, the medical disease is always the strongest, and must win in this struggle for existence; it utterly ignores the sick body, which is the "bone of contention" in the fight.

In this statement may be found the secret of the non-acceptation of homœopathy by veterinary medicine. Animals with more sense than human beings resented being subjected to such vivisectional cruelty, one disease being enough for them without permitting man to cause another at the same time. That two diseases cannot exist together at the same time in the same organism, whether as the result of medicinal poisoning or not, is one of the greatest fallacies that an ignorant and unscrupulous organization ever sought to drive into the brains of a credulous public. Daily experiences so frequently prove the absurdity of such a hypothesis, that argument is unnecessary. To be sure, a few disgruntled, unsuccessful, or erratic members of the veterinary profession have advertised themselves as homœopaths; still fewer, men of no reputation whatsoever, have advertised that "they would practice either as homœopaths or allopaths, according to the will and pleasure of their patrons." Not every one that the public allows to dub himself "veterinary surgeon," has a claim to the title.

Homœopathy dares not deny that it has practically died out, and that it has neither recognized standing nor respectability in the land of its nativity, Germany. It dares not assert that it has taken any active part in the great movements by which medical knowledge has been advanced in this century, especially in France during the first, and Germany in the last half. When we glance over the scroll of honor, and read the names of those men that, irrespective of any "ism," have made medicine what it is to-day by the devotion of their lives and energies to original research, we look in vain for that of a single homœopath. They are conspicuous only by their absence. When we ask who have been the founders of modern Pathology and Pathological Anatomy—the very foundation of our knowledge of disease—we are told, Bichat, Rokitansky, Virchow, and their immediate students. If, on the other hand, we ask who have laid the foundation of our modern Anatomy, Physiology, and Histology, we are told, Magendie, Claude Bernard, Du Bois Raymond, Henle, Rindfleisch, Frey, Waldeyer, Huxley, Gegenbauer, Krause, Foster, Brücke, Pettigrew, Ludwig, and others.

It is no wonder, however, that homœopathy has taken no part in advancing our anatomical knowledge by research. Our astonishment would be increased to know that they even teach it at all, when one of its greatest lights in its apostolic days wrote that "children in the school learn as much anatomy as is necessary for one to know in order to cure diseases. Should one, however, desire to extend his knowledge of this subject, he can watch a butcher slaughtering swine. Whoever believes that he requires more anatomy in order to effect cures, makes a mistake."—(*Lutze.*)

After reading the above, it cannot certainly surprise us to know that homœopathy never has had, and has not to-day, any surgery. Every one of the methods used for operations in its hospitals have been appropriated from those outside barbarians whom they christen the "old school." Homœopathy has yet to present the world with a man like Lister, Langrubeck, Billroth, Gross, Sayre, Marion Sims, or a Spencer Wells. "But surely they must have helped advance clinical medicine, they are so successful in practice, you know?" Unfortunately for all such systems, or in fact for any, a doctor's real reputation—the one that lives after him—does not depend upon his antemortem success in making practice and money, or pleasing people, but rather upon the evidence that he has given to the world—the mark he leaves behind him—that he knew something, that the world was richer in knowledge for his having lived.

Homœopathy knows nothing with reference to the prevention of disease. It is therefore not surprising that it has taken no part in this the most modern step by which scientific medicine notes its onward march. Again we look in vain for the names of homœopaths, and in their stead we read of Pasteur, Koch, Klein, and others. In this regard, even that despised stem, Veterinary Medicine, may hold up its head with pride, and recount the names of Gerlach, Hering, Furstenburg, Hertwig, Colin, Bouley, Toussaint, Chauveau, Feser, Schütz, and others in Europe; while America may remind them of a Salmon and a Law. As we have said, Homœopathy has taken no part whatever in these great works; it has been too busy making money. Within the last few years, however, its leaders have had brains enough to appropriate the results obtained by these "old school" laborers. Homœopathy is a parasite that draws its vitality from the modern school of medicine. The "old" has become the "new," and the "new" the "old school" of medicine. Homœopathy has no conscience! It can always give something. This is its stronghold. Its self-dispensing system is never at a loss for a remedy for body or mind. Homœopathy does not essay to treat disease. It cures symptoms. Its remedies are specifics for this or that. For a remedy to be a specific, necessitates that it be a positive cure in every case. To cure, means to reinstate a diseased part in the same condition it was before being diseased. There must not be the slightest change in its tissues from absolute normality, or a particle of weakness or liability to secondary attacks left.

Nothing can occur without cause. Homœopathy came into existence at a period of inanition in German medicine. Everything was dead, or sleeping a deceptive sleep. The brilliant work of the great French masters of the latter part of the last century and first of this, would not, naturally, be very willingly accepted by a subjected people. As it was, it passed into Germany by the way of the Vienna school, Rokitansky, Skoda, and others being the means. At this time there was no research, no scientific method, at the German schools. "Alles war bei dem Alten." Horrible mixtures and worse advice were the rule. In the midst of this apparent death came a reaction, or better, an irruption. This was Homœopathy. A recent writer, Koeppe, thus describes the conditions in Germany at this time:

"The end of the 18th and beginning of the 19th century marks a peculiar epoch in the history of medicine. The past had left nothing of value behind with reference to the knowledge and treatment of inner diseases; there existed nothing but a chaos of raw empiricism and wilful deductions, which called forth the ridicule of a Moliere and Hogarth. While in other lands men were endeavoring to provide a solid foundation for the future development of scientific medicine, the greatest uncertainty and crudeness prevailed in Germany. The blame for these unenviable conditions must be sought in the influence of the speculative direction which then ruled in German philosophy. No one thought to reckon with concrete facts; all tendency to cold-blooded, scientific observations failed; genuine investigations were neglected, or when made, found no consideration on account of the noise made by the philosophical disputants. Chemistry, physics, physiology, and histology were scarcely begun, and pathology busied itself with abstruse conceptions. Authors amused themselves with the construction of innumerable theories and systems, which were as poor in positive results as they were rich in hypotheses and errors."

It is to the credit of Samuel Hahnemann—born at Meissen, 1755; died at Paris, 1843—the founder and author of Homœopathy, and to the polemic vigor with which he defended his views, that German medicine awoke from its self-complacency. The German doctors really knew not what to say; they stood with brains asleep and mouths agape while this bold interloper and his immediate followers disturbed not only their serenity of mind but their incomes as well. He called them the "Old School," and finally "Allopaths"; his followers were known as the "New School," and his teachings as "Homœopathy." He made so much noise, the others saying nothing, that his fame spread very rapidly. The "new" beat the "old school" out of the field. "New brooms sweep clean," at first. Hahnemann had something to offer besides drugs. He came with promises—promises to cure. Come to me, all ye that are sick; take but of the medicine that I offer you—it is sweet, tasteless, and harmless, and costs nothing—and ye shall be cured! No wonder that they came. Out of the darkness of routinism they beheld the rising sun of homœopathy, which promised to drive away their ills as mists before the morning sun. My system is "Gottes Gabe"—a gift from God—he told them. The multitude believed, and flocked to him and his followers, as they did to Jesus and His Apostles of old. What did he give them?

Hahnemann was an enthusiast. If he had not believed in himself as one inspired of God, as a prophet of the truth—that God had opened to his mind the keys to health—he had not been able to impress himself and his system so strongly upon others. His system is the only one, out of many, that has had vitality enough to stand for nearly a century in opposition to the regular school. It must have had something in it but error. It had. We should not forget, however, that many "false prophets" have arisen both before and since Hahnemann, each of whom claimed that either they or their teachings were "gifts from God." That cry is always taking. It always draws followers, no matter by whom uttered. If the "gift," however, is all they have to offer, the prophet soon finds himself without honor in his own country. It is because Hahnemann had something more than the "gift" to offer, that while the "gift" is of no value, still the system has endured.

In the first place, homœopathy paid great attention to the externals and surroundings of its patients. Its peculiar tenets made it absolutely necessary that no disturbing influence should come near the patient. The greatest attention was given to hygiene and diet. Many absurd regulations were made in this direction, none of which were more so than those with relation to the injurious medicinal action of cooking salt as ordinarily used. If strychnine were only one-half as dangerous as the homœopaths would have us believe common salt to be, the world would have been depopulated long since. But above everything else, the most valuable thing Hahnemann did for the Germans was to teach them that "cleanliness is next to godliness," and that water has some other uses in this world than for fishes to swim in, or ships to float on, or even to make beer out of. Hahnemann did even more, he taught the world the hitherto unknown fact that medicines must be used with some respect for the organism they were to be offered to. While "provings" upon the healthy organism are not beyond questionable therapeutic value, being often but suggestive, still it cannot be denied that homœopathy indirectly led to the birth of the scientific method in therapeutical research. Prove all things *on yourself*, seems to have been Hahnemann's motto. We stick to the first part, "prove all things," but have little respect for "self-provings." While we cannot claim for Hahnemann *all* the credit for awakening German medicine from its comatose condition, still there is no doubt that he was a thorn in its side that hastened along the work.

It is singular that homœopathy took no part in this advance, as we have already shown; but when we come to study the fundamental principles of this system, we shall be surprised that even latter-day homœopaths have consented to adopt the results. Homœopathy undoubtedly hastened the birth and development of the child that strangled her to death in Germany, and that will yet do it all over the world. "The scientific method in every branch of medical research," is the somewhat long name by which this child is known. The effect of homœopathy in helping along this great work fully proves the fact that an erroneous or false theory or principle, ably and polemically defended, is of more value to the world than any amount of truth wanting earnest defenders. Should future developments prove the theories of Darwin to be erroneous, still history will say that no one man that has ever lived has given the same amount of stimulus to every variety of scientific research; nor has any one man's work ever led to so many valuable discoveries, to the unveiling of so many "connecting links" in the line of vital development, as the work of Darwin.

Modern medicine has contributed its part to this grand work. It accepts nothing as true on the weight of authority only. It has learned that many things which man has held true for centuries, have not stood the test of modern methods of research. It considers no question as so settled that it cannot be again a matter of open discussion. Its method is one of continued and interrupted provings. New discoveries become the tests by which old truths are proved. It subjects the crucible of the chemist, the scales and measures of the physicist, the scalpel and microscope of the anatomist and pathologist, and even the animal world, to the physiologist and etiological student in its great search after the exact facts. It knows no "ism," no "pathy." Its spirit is as free as the driving wind. "It goeth wheresoe'er it listeth," and nothing is sacred from its inquiring eyes. It has no sympathy with error, no matter by whom uttered. It respects the man, but not his errors. A Virchow is no more sacred from attack than the veriest tyro in its laboratories. The ancient uttered its motto when he said: "Truth is the mightiest of all things; it alone can live forever." The spirit of modern medicine is ever active. It is also aggressive. It does not point to its past, and with self-complacency say to the questioner, "See what I have done." In an obtrusive and positive manner its work says to the world, "See what I am doing."

Homœopathy suffered somewhat from its offshoots at a very early day in its history. The most creditable of these is known as the Nihilistic School. Skoda was its chief light. The brilliant results which followed the workings of homœopathy, in comparison with the opposing school of medicine, were attributed by Skoda to be entirely due to the almost religious exactness with which the hygienic and dietetic regulation of patients was carried out. Skoda was right, whether with reference to homœopathy or any other school of medicine, when he attributed much to these things, but wrong when he denied all value to medicines in any form.

An erratic child—which was but a natural outgrowth from Homœopathy, on the principle that one extreme is often followed by another—is known as Isopathy. The advocates of this

"ism" asserted that to cure any disease, it was necessary that the individual diseased should be treated either with original portions of the same disease, or preparations from them. So we find tuberculous lung material given for phthisis; the discharges of gonorrhœa and syphilis, or preparations of the same, for those diseases; diseased livers, hearts, kidneys, etc., for diseases of those organs. Such a disgusting system of therapeutics could not naturally long continue without suffering modifications, and so we find that instead of the products of disease, or preparations from them, the Isopaths soon gave the healthy organ from some animal to cure diseases of similar organs in man. The organs of the fox were supposed to have an especial value in this direction.

The corner-stone, the very foundation of homœopathy, upon which all its apostles and followers agree in public, whatsoever their private thoughts may be, is the "indicatio remedii in symptoma similia," or "similia similibus curantur" (like cures like), which Hahnemann asserted to be directly opposite to the principles of the "old school," which he expressed to be "contraria contrariis curantur," or that diseases were always to be treated "per contra."

Neither one of these principles can be called a therapeutical law. There can be no law for the treatment of disease, though human experience has long since shown that remedies of a "similar" nature are sometimes applicable to certain disturbances. For instance, we apply cold to frost-bites, heat to burns; but this principle cannot be generalized. The modern school of medicine knows no universal law of therapeutics, any more than it recognizes a law of disease. The laws of disease and those of health are one, although homœopaths have utterly ignored their study in the past, whatever effect modern research may have made upon their principles. Homœopathy resembles Religion again, or better, Christianity, in that we must not go to the new school of homœopaths to find out what it is. It is founded upon principles that were taught them by one man and his immediate followers, so that to find out what they really are, we must go to the "Organon and writings of Hahnemann and his pupils," in the same manner that one who desires to know what Christianity is, should go to the New Testament, and not to the writings of the sects, that are somewhat founded on them. That our conclusions are correct, is more than sufficiently shown by the following quotations :

"Although the study of anatomy may serve pathology, and has advanced the latter—good ; by means of pathology we may learn what disease is, but cannot learn how to cure it, which is the all-important thing. All that we need is an exact conception of the symptoms of a disease, they being the answer of nature to our demands, in order to surely cure."

"Has physiology any value to the doctor in relation to the curing of disease ?"

"Curing alone is the most important task of the doctor, and that which does not advance this art is useless ; wherefore shall the doctor waste valuable time in the study of such useless things ? Hahnemann recognized this fact, and made the solving of the task of curing, his life-work."

"Heim was another that recognized this as the all-important duty of the doctor. When any one spoke to him about pathology or physiology, he laughed, because he knew that these things are not able to cure, even a finger. His practical common-sense, his correct physician's instinct, ruled him in all his undertakings. If any one asked him 'Why ?' he answered, 'Bah, I don't know that. Do as I tell you, and you will be cured'; *and it always happened as he said.*"

"It was given to Hahnemann to discover the secrets of nature and the law which is as eternal as the world, and according to this uncontrovertible law, to heal disease surely, quietly, and with gentleness."—(*Lutze.*)

I have already said that "the laws of health and disease are the same." The laws do not change when disease attacks any portion of an organism. It is the action of the elements composing the diseased parts that changes. They no longer act physiologically, i. e., normally. Hence, to know what disease is, it is absolutely necessary for the doctor to know what the physiological or normal action of an organ is, Hahnemann to the contrary notwithstanding. The action of the elements of any organ can only depart from normality in one of three directions; there must either be a plus, a minus, or a cessation of action altogether to constitute disease. If either of these changes occur in an organ of vital dignity, death soon results ; but if they take place in one of less importance, they may continue for a variable length of time, and still restitution occur. Hence the necessity of the doctor's being intimately acquainted not only with anatomical seat, but the physiological action and importance of every part of the body. "Similia similibus curantur."

The foundation of homœopathy is dependent upon personal observations with reference to their remedies made upon the person's self. Such observations can never be trusted. Every person of any observational power knows what the effects of imagination are with reference to disease in themselves. If we are among diphtheritic patients in the early days of hospital life, we are continually having pains in the throat; if in the pneumonia ward, pulmonary complaints rob us of sleep; if studying nervous diseases, softening of the brain, epostic paralysis, or something else frequently drive many a young student to his teacher. Patients are continually coming to us, thinking they have "Bright's" or some kindred trouble. When we ask "Why?" Because of a "pain in the back," which less indulgence of the sexual and other appetites would soon relieve.

Hahnemann deduced his special therapeutical law from certain experiments made upon himself with a preparation of quinia bark. He prepared a decoction from one ounce of bark in two pounds of water. This he drank very gradually until used up. He affirmed that the preparation induced an attack of fever; hence "China" is a specific for fever. Both homœopaths and allopaths have since repeated the same, or similar experiments, even to very large doses of quinine, but fever has never resulted. The principle that "like cures like," is the keystone of homœopathy. Let it speak for itself. "In order to effect a speedy, gentle, and certain cure, it is only necessary for the doctor to select a remedy which can itself excite a similar disease to the one he desires to cure."

"The real nature of disease is hidden from us. All we can discover is the symptoms which represent the disease."

"As inner changes are in no way ascertainable, and all that we can see are the symptoms of a disease, it is the duty of the doctor to consider the latter only; when he has caused them to disappear, the disease is cured" (*sic*).

"The eternal and general natural law, 'similia similibus curantur,' rests upon the axiom—what is an axiom?—that only one disease can exist in an organism at the same time. The second disease, the medical disease, MUST be stronger than the first—an exacerbation of the homœopathic brain—and it is but natural that the latter should be overcome. In this way an acute disease may be cured by another of the same nature, or a chronic disease by an acute."

"That is the only correct and curing remedy for every diseased condition which can produce a similar condition in a healthy organism."

"An artificial disease will be produced by the remedy which resembles the natural disease, and which will drive the latter away."

"As soon as the axiom was discovered that a medicine could only be the right one in a given case when it was capable of causing a similar disease in a healthy person, it at once became the imperative duty of the homœopaths to prove their remedies."

"This caused an entire change in the views of people as to what disease really is."

"The sum total of the symptoms forms the disease."

"The homœopathic doctor will not, and does not need to know anything further than the whole of the symptoms and the ascertainable next causes, such as cold, heat, shock, anger, etc., in order to cure disease; for when the symptoms disappear, the disease is cured."

"So long as a single symptom of disease remains, the latter is not cured."

These quotations, which could be indefinitely multiplied, not only from the Organon, but from all the early authors upon homœopathy, sufficiently show the character and depth of that system. In the light of modern scientific research, they so completely show the shallowness of the whole system that criticism would be like trying to blow out a soap bubble; before one got ready, the bubble might burst. That two diseases cannot exist in one and the same person at the same time, is so often contradicted by daily experience as to need no further notice. That a medicine can cause a disease, in medicinal doses, is too ridiculous for any sane person to consider. According to homœopathy, however, the medicine does not cause the same disease when taken by a healthy person, but similar symptoms. Homœopathy knows nothing about disease, as we have shown. Symptoms are a teleological creation of an all-beneficent Father, made especially for the benefit of homœopathic physicians. To us they bespeak internal conditions, many of which all the remedies in the pharmacies in the world cannot cure, either directly or indirectly. It is a well known fact that Hahnemann went all to pieces in his work on chronic diseases, which fully demonstrates that though symptoms could disappear, such diseases could not be cured by homœopathy. A disease which this principle of homœopathy has been completely wrecked upon is syphilis, the entire visible symptoms of which may disappear for years, and yet tertiary developments occur in course of time. Hahnemann ridiculed the idea that the doctor could know anything of disease itself. Wunderlich says that "he constantly avoided being present at autopsies." The latter-day saints of this medical sect have, however, seen fit to borrow the results of all the studies upon this subject that have been made in the laboratories of the "old school." "Similia similibus curantur," or in other words, to drive one devil out of a man we introduce another of a "similar" nature. It is almost an insult to the intelligence of humanity to think that they could accept such nonsense, even for an instant. "Similia similibus curantur." If a man is drunk on whiskey give him rum, would be an equally sensible argument. If a patient has an embolic pneumonia, pour oil, quicksilver, or some medicine into his jugulars. The fundamental principle of homœopathy is in direct contradiction to human intelligence and every result of modern research. It bears upon its face such evidence of error that every person capable of a logical use of his reflective powers should at once see its utter fallacy.

HOMŒOPATHIC THERAPEUTICS.

If there is no law for disease, there certainly can be none for the preparation of drugs such as the "Dilution Theory of Homœopathy," *no remedy has but one specific action.* Specifics are the next thing to humbugs. Homœopathy does not recognize the variable actions of medicines. It says, because "quinia will cause fever, it will cure it." It does not prove that it will cause it. Quinia not only has its chief and well known antipyretic action, but also has its almost specific anti-germ action, which cannot be got in small doses; a sufficient saturation of the blood to check the further development of germs is necessary to its successful use in such cases. Quinine, again, has its tonic action in small doses. All of which are not described by Hahnemann. Homœopathy has no "expectative treatment"; it knows nothing but the *specific action of drugs.* It has taught us to simplify the ancient mixtures; but when it tells us that there is a "dynamic force," an invisible spirit, in medicine, that is let out or freed by "shaking and rubbing," it insults our intelligence. Medicines will only act in certain well known quantities. The question is to decide by actual observations and experiment what these quantities should be; below a given quantity, even the most powerful poisons have no action. Homœopathy does not recognize the cumulative action of certain remedies. But we will let it speak for itself:

"It is impossible to separate the small doses of highly potentized remedies from the doctrines of homœopathy."

"That a small, even the smallest, dose of a remedy can produce the most wonderful changes in the normal, healthy body, is abundantly proven in Hahnemann's 'pure materia medica.' It proves that in the excited conditions of disease, a much stronger, often too strong an action of the remedy may result, which may be followed by injurious effects. Hahnemann experienced this almost daily, and he came upon a method of lessening the violent action of remedies, not by the nonsensical offering with them of correctives, as is done by the 'old school,' but by means of dilutions of the pure substances with indifferent materials, such as pure alcohol, or water, or by trituration with milk-sugar."

"He taught us that we must press out the fresh juices of vegetable remedies (he discarded the results of chemistry by which the active principle of drugs is separated and simplified for our use. In this regard let me say that I recently met an allopathically educated homœopathic doctor, who informed me that he had just begun to use morphine, and found it very good in the third trituration!); these juices he mixed half and half with pure alcohol, and allowed them to remain in closely stoppered bottles until clear; the clear fluid is then to be poured off. The alcohol prevents all fermentation or change in the remedy, and it retains its full power when kept away from the light and is securely corked. Such preparations form the 'Uhr' or 'mother tinctures' of Homœopathy."

Insoluble stuffs from the mineral kingdom are to be won by trituration, in that we take a grain of the mineral and rub it in a mortar with 100 grains of sugar of milk for an hour. From this preparation, one grain is again taken out and rubbed in the same manner for one hour with another 100 grains of milk-sugar, and from this another similar amount is to be rubbed for an hour with another 100 grains of milk-sugar, the three triturations giving a soluble preparation. Of this third trituration, one grain is to be added to 100 drops of pure spirits and water, and shaken.

Hahnemann diluted the mother tinctures, already spoken of, by adding one drop of these tinctures to 100 drops of spirits or water, and by means of vigorous shaking thus united the medicine with the constituents. From this preparation, one drop is again to be taken and added to 100 drops of spirits or water, and vigorously shaken; this procedure is carried on until the thirtieth potency is obtained, which will contain one-decillionth of a drop of the 'mother' tincture. With one drop of this latter potency, 500 to 1,000 pellets are to be moistened (?), and the medicine is ready. Of these, "one is to be placed upon the tongue, or dissolved in water, or only smelt of," etc.—(*Lutze.*)

Homœopaths have endeavored to find proof for the dilution theory in the action of germs in the infectious diseases known to be due them. They could not have fallen upon a more shaky foundation. No remedies, known to either homœopathy or any other system of medicine, have the power of auto-multiplication within the organism into which they are introduced. Specific quantities are not necessary to specific action of germs. A single bacillus might, under favorable conditions, produce anthrax. The cumulative action of certain drugs is not due to the multiplication of their elements in the organism, as is the case with germs, but because the organs do not eliminate them as rapidly as they are introduced; hence their accumulation, until poisonous symptoms appear, but only when the quantity thus accumulated has become a specific amount.

### CONCLUSION.

We have endeavored to truthfully illustrate the two great principles of homœopathy. We have no idea that our deductions will be acceptable to homœopaths. We have conclusively shown that there can be no law of therapeutics, and that "similia similibus" is as senseless and practically illogical as "contraria contrariis curantur." We have shown that the dilution theory is as arrant an illusion as was ever forced upon the unreflecting brains of an innocent humanity. We have shown that no medicine has one exclusive specific action, while many have specific employment in therapeutics; still they have other actions that are of scarcely secondary importance. The poisonous action which certain drugs have is no indication of their therapeutic value. To say that mercury is, what it nearly approaches, a specific in syphilis, because if given until cumulation has occurred, ulcerations, etc., follow, is an error; it is its alterative action of which we take advantage.

Many medicines have chemical affinities with certain tissues, but contrary to the assertion of homœopathy, these affinities exist for those tissues wherever they occur in the living organism. There is no such thing as a science of medicine. Medicine is the result of the application, first, of the experience of the crude intellectual observations of developing man to the curing of disease. Ancient and mediæval medicine was entirely curative in its endeavors. Homœopathy is the same. With the development of man, medicine also advanced. Modern medicine is the application of the results of the endeavors of scientists to the treatment *and prevention of disease.* We have shown that homœopathy has taken no part in this work.

It is with the advocates of such a system of errors, misconceptions, false promises, and superstitions that these "new codists" would have us associate in a collegiate manner. The thing is a logical impossibility, if we would be true to that spirit which has made modern medicine one of the glories of the nineteenth century. This spirit is one of untiring research, and is directly opposed to that of homœopathy, which has ever been true to itself, in that, as we have shown, it has utterly ignored research. It will not do for them to say, "But we have adopted its results," when they have taken no part in it. Water and oil have as much affinity for one another as there exists sympathy of purpose between homœopathy and modern medicine. Homœopathy is to-day, and always has been, a dealer in specifics and a promiser of cures.

While modern medicine recognizes the imperative necessity of treatment, and honestly endeavors to scientifically apply the results of therapeutical research to that end, *still its greatest aim is the prevention of disease.* Homœopathy shows its utter disregard of human welfare by having totally neglected to take part in or contribute to this great work. Homœopathy's greatest endeavor is, and has been, to fill its own pockets to repletion, while it as successfully depleted those of a confiding humanity. The motto of modern medicine is "Onward." Progress marks its pathway. The eyes of Homœopathy are directed backward. It has but one god, and that is Hahnemann; and the Organon is its book of revelations. Modern medicine is characterized by the free spirit of inquiry; while homœopathy still struggles in the ruts and byways cut out by Hahnemann a century ago.

We must not seek to meet this enemy of progress by argument alone. Its honeyed words and sweeter remedies have still too great a hold upon the minds of a humanity which is still as a "babe in swaddling clothes," so far as its intellect is developed, or it has acquired any knowledge of its own being. The unanswerable argument which the comparison of the work done by the two schools shows, needs, however, frequent repetition. But we must keep alive this spirit for work. Germany has shown us the way. We can see the result.

Let us then all hasten the day when American medicine shall be equally characterized by this spirit of research, and when investigation shall become as important a part of the work of medical schools as teaching is at present. When that day comes, homœopathy will have become a thing of the past, and American medicine will know but one profession, one spirit, one purpose. Until that day there can be no union of the schools. *Truth has no sympathy with error, nor science with charlatanry.*

